# High-Density CRISPR/Cas12a-Mediated Multiplex Genome Editing Reveals Genome Instability in Allotetraploid Cotton

**DOI:** 10.3390/genes17060622

**Published:** 2026-05-29

**Authors:** Chuanying Zhu, Yaxin Wang, Mingjv Zhu, Gefei Chen, Fuqiu Wang, Bo Li, Zhongping Xu, Guanying Wang, Jinchen Xu, Xinzimo Lu, Yanqin Wang, Shuangxia Jin

**Affiliations:** 1 Xinjiang Production & Construction Corps Key Laboratory of Protection and Utilization of Biological Resources in Tarim Basin, College of Life Sciences and Technology, Tarim University, Alar 843300, China; zhucy3516@gmail.com (C.Z.); 107572023417@stumail.taru.edu.cn (J.X.); xinzimolu@hotmail.com (X.L.); 2 State Key Laboratory of Vegetable Biobreeding, Institute of Vegetables and Flowers, Chinese Academy of Agricultural Sciences, Beijing 100081, China; wangyaxin@caas.cn; 3 Key Laboratory of Biology and Genetic Improvement of Flower Crops (North China), Ministry of Agriculture and Rural Affairs, Beijing 100081, China; 4 Hubei Hongshan Laboratory, Huazhong Agricultural University, Wuhan 430070, Chinachengefei1222@163.com (G.C.); xzp@mail.hzau.edu.cn (Z.X.); gyw@mail.hzau.edu.cn (G.W.); 5 College of Agriculture and Biology, Liaocheng University, Liaocheng 252000, China; wangfuqiu@lcu.edu.cn; 6 Xinjiang Key Laboratory of Crop Biotechnology, Institute of Nuclear and Biological Technology, Xinjiang Academy of Agricultural Sciences, Urumqi 830091, China; libo@xaas.ac.cn

**Keywords:** cotton, CRISPR/Cas12a, multiplex genome editing, high-density editing, genome stability

## Abstract

Background: Upland cotton (*Gossypium hirsutum*) is a major natural fiber crop and an important model for studying genome evolution and gene function in polyploid plants. However, its large and highly redundant genome presents substantial challenges for efficient and coordinated multiplex genome editing. Methods: Here, we developed a high-efficiency CRISPR/Cas12a-based multiplex genome editing system in cotton by integrating a tRNA–crRNA polycistronic expression strategy with a Bean yellow dwarf virus (BeYDV)-derived replicon. Results: This platform enabled coordinated expression of multiple crRNAs and simultaneous targeting of 16 loci within a centromere-proximal region of chromosome D03 (18.65–24.47 Mb). In individual transgenic lines, up to 10 target sites were edited concurrently, with nine targets exhibiting editing efficiencies above 56% and the highest efficiency reaching 96.46%. High-density multiplex editing predominantly induced small insertions and deletions at target loci. Notably, edited plants exhibited reduced growth and pronounced cytological abnormalities, including chromosome bridges, lagging chromosomes, and abnormal meiotic products. Transcriptome analysis revealed widespread dysregulation of genes involved in chromosome segregation and cell cycle regulation. Despite these functional perturbations, HiFi long-read sequencing detected no large-scale chromosomal rearrangements, indicating that genome instability arises from cumulative local perturbations rather than global structural alterations. Conclusions: Together, our results establish an efficient multiplex genome editing platform in cotton and highlight potential constraints of high-density editing on genome stability in complex plant genomes.

## 1. Introduction

As a typical allotetraploid species, upland cotton (*Gossypium hirsutum*) possesses a large (~2.5 Gb) and highly complex genome characterized by extensive gene redundancy, abundant repetitive sequences, and intricate regulatory networks [1,2,3,4]. These features pose substantial challenges for functional genomics studies and precise genetic improvement, particularly when targeting gene clusters or non-coding regulatory regions.

The emergence of CRISPR/Cas-based genome editing technologies has revolutionized plant functional genomics and molecular breeding [5,6]. Among them, CRISPR/Cas9 has been widely applied for targeted mutagenesis and chromosomal engineering, including multi-target editing [6,7,8]. In parallel, CRISPR/Cas12a (Cpf1) has gained increasing attention due to its unique features, such as the ability to process crRNA arrays, recognition of T-rich PAM sequences, and generation of staggered DNA ends [9,10]. These properties make Cas12a particularly suitable for multiplex genome editing, enabling simultaneous targeting of multiple loci within the genome. Despite these advances, most studies in plants have been limited to editing a small number of targets, and efficient strategies for high-density multiplex editing, especially in complex polyploid genomes, remain underdeveloped.

Geminiviruses are a large family of plant-infecting DNA viruses characterized by their rapid infection, replication, transcription, and expression without the need for integration into the host genome [11]. This feature makes them ideal vectors for transient expression and genome editing applications, as they can produce abundant transcripts to enhance editing efficiency within plant cells [12]. Geminiviruses typically possess either one (monopartite) or two (bipartite) single-stranded circular DNA components (2.5–3.0 kb), encoding four to eight proteins that mediate viral replication, movement, transmission, and pathogenicity [13,14]. A Bean yellow dwarf virus (BeYDV)-based replicon system has been successfully developed for high-level heterologous protein expression in plant cells [12]. BeYDV replication depends on three viral elements: the cis-acting long intergenic region (LIR), the short intergenic region (SIR), and the trans-acting replication initiation proteins (Rep/RepA) [12,15]. To adapt the BeYDV genome as an editing vector, the coding sequences for movement and coat proteins were replaced with site-specific nucleases and donor templates, while LIR sequences were positioned on both sides of the construct to facilitate T-DNA release within the plant nucleus. The BeYDV replicon fragment was incorporated between the left and right border sequences of an Agrobacterium tumefaciens Ti plasmid vector. During Agrobacterium-mediated transformation, the T-DNA carrying the BeYDV elements was transferred into cotton cells, where circular replicons were released and autonomously amplified through rolling-circle replication [16]. This replication mechanism can generate hundreds to thousands of copies of gRNA and donor molecules per cell, thereby substantially increasing the local concentration of editing components and greatly improving the overall genome editing efficiency.

In addition to technical challenges, the biological consequences of high-density genome editing are still poorly understood. Simultaneous induction of multiple double-strand breaks (DSBs) may impose substantial stress on genome integrity, potentially leading to chromosomal instability, abnormal cell division, and altered gene expression [17]. Although genome editing is generally considered precise at the sequence level, its impact on higher-order genome organization and cellular processes has not been systematically investigated, particularly in polyploid crops.

In this study, we developed a high-efficiency CRISPR/Cas12a-based multiplex genome editing system in cotton by integrating a tRNA–crRNA polycistronic (PTC) processing strategy with a Bean yellow dwarf virus (BeYDV)-derived replicon. This system enables the simultaneous expression of up to 16 crRNAs and achieves high-density genome editing in a polyploid background. Using barcode sequencing and PacBio HiFi long-read sequencing, we systematically evaluated editing efficiency across multiple target sites. Furthermore, we investigated the phenotypic, cytological, and transcriptomic consequences of high-density editing, revealing its potential impact on genome stability and cell division. Our findings provide new insights into the limits and biological effects of multiplex genome editing and offer a powerful tool for functional genomics and genome engineering in complex crop genomes.

## 2. Materials and Methods

### 2.1. Plant Materials and Growth Conditions

Upland cotton (*Gossypium hirsutum* Jin668) was used as the experimental material. Seeds were germinated and grown under controlled greenhouse conditions (28 °C, 16 h light/8 h dark photoperiod, 60% relative humidity). Transgenic plants were generated via Agrobacterium-mediated transformation as described below. Non-transgenic Jin668 plants regenerated through tissue culture were used as wild-type controls.

### 2.2. Design and Construction of High-Density Multiplex CRISPR/Cas12a System

Based on the pBeYDV-Cas9 vector construction method described by Li et al. [1], we synthesized the rolling circle replication elements of the geminivirus (ULIR, SIR, REP, and DLIR) in two segments through a commercial provider (GenScript, Nanjing, China). The ULIR element was synthesized independently, while the SIR, REP, and DLIR elements were fused into a single continuous segment (SIR-Rep-DLIR). The original LbCpf vector was digested with the BsaI restriction enzyme, and the ULIR element was inserted upstream of the GhU6-7 promoter via In-Fusion cloning. Simultaneously, the SIR-Rep-DLIR composite segment was incorporated downstream of the crRNA cassette to obtain pBeYDV-LbCpf1 vector (17.723 kb).

Sixteen target sites on chromosome D03 (18,647,084–24,468,261 bp) were selected using an online CRISPR target design tool (http://skl.scau.edu.cn/targetdesign/ (accessed on 6 April 2023)), based on the T2T reference genome of upland cotton [3]. Synthetic crRNA sequences were assembled into a tRNA–crRNA polycistronic (PTC) array and cloned into the pBeYDV-LbCpf1 vector using In-Fusion cloning technology, generating the multiplex editing construct pBeYDV-LbCpf1-D03T16 (19.737 kb). Cas12a expression was driven by a constitutive ubiquitin promoter, while a BeYDV-derived replicon was incorporated to amplify intracellular template abundance, thereby enhancing crRNA expression and overall editing efficiency.

### 2.3. Agrobacterium-Mediated Transformation and Selection

The pBeYDV-LbCpf1-D03T16 vectors were introduced into *Agrobacterium tumefaciens* strain GV3101 by electroporation. Hypocotyl explants derived from Jin668 seedlings were transformed according to previously established protocols in our laboratory [18,19]. Briefly, after surface sterilization, seeds were incubated in the dark at 30 °C for 5–7 days. Hypocotyls were excised into 5–7 mm segments and used as explants for Agrobacterium-mediated transformation. Approximately 500 hypocotyl explants were used for transformation. Transformed tissues were cultured on selective regeneration medium supplemented with kanamycin (50 mg/L). Following selection and regeneration, four regenerated plants were obtained and subsequently transferred to soil for greenhouse cultivation. PCR-based genotyping and barcode amplicon sequencing were performed to confirm the presence of the CRISPR/Cas12a construct and identify successfully edited transgenic lines. Ultimately, four independent edited lines were obtained for subsequent analyses.

### 2.4. Detection of Transgenic Materials

Samples were collected from callus and regenerated plants for DNA extraction. Total DNA was extracted using the CTAB method, and positive transformants were identified via PCR using Cpf1 protein-specific primers. The primers were designed with Primer Premier 5 software and are listed in Supplementary Table S1.

### 2.5. Detection of Genomic Target Site Editing

The target editing efficiency was assessed using Sanger sequencing and barcode sequencing. To ensure compatibility between Sanger sequencing and high-throughput sequencing, primers were designed to amplify fragments no longer than 300 bp in the cotton genome. Given that gene editing systems can introduce a high frequency of indels at target sites, primers should not be positioned too close to the target site. Instead, a minimum distance of 50 bp should be maintained between the primers and the target site to accommodate potential sequence variations. Barcode amplicon sequencing data were analyzed using CRISPResso2 software to identify mutation types and quantify ed-iting frequencies at each target site. Editing efficiencies were calculated as the propor-tion of sequencing reads containing insertions, deletions, or substitutions relative to the total number of mapped reads spanning the target regions. The primers for target site editing detection are listed in Supplementary Table S2.

### 2.6. Phenotypic Effects of High-Density Multiplex Editing on Chromosome D03 in Cotton

To assess the phenotypic consequences of high-density multiplex genome editing on chromosome D03, transgenic cotton plants harboring a CRISPR/Cpf1 construct targeting 16 loci were grown under controlled greenhouse conditions, with non-transgenic Jin668 plants serving as wild-type controls.

For phenotypic documentation, a representative T0 transgenic line was selected for detailed imaging. At key developmental stages, whole plants, flowers, and mature cotton bolls were visually inspected and photographed using a high-resolution digital camera. Images were captured under standardized lighting and background conditions to facilitate accurate morphological comparison. Representative phenotypes were documented, and floral organs, including petals, anthers, and stigmas, were examined for size, symmetry, and pigmentation differences relative to the wild type.

### 2.7. Cytological Observation of Mitosis and Meiosis

To assess the cytological effects of multiplex genome editing on cell division, root tips and anthers were collected from both edited and wild-type cotton plants. For mitotic analysis, root tips (1–2 cm) from 24-h-old seedlings were pretreated with saturated α-bromonaphthalene at 4°C for 3 h, fixed in Carnoy’s solution (ethanol: acetic acid = 3:1) for 24 h, and subsequently hydrolyzed in 1 N HCl at 60 °C for 10 min. The samples were then stained with 2% aceto-carmine and gently squashed to prepare chromosome spreads.

For meiotic analysis, floral buds (2–3 mm) at suitable stages were dissected, and anthers were squashed in 1% acetocarmine. Meiotic stages from diakinesis to tetrad formation were examined under a light microscope. Abnormalities such as lagging chromosomes, chromosome bridges, micronuclei, and irregular tetrads were recorded. Images were captured using a Leica DM5000B microscope (Leica Microsystems, Wetzlar, Germany) with a CCD camera. Data were statistically analyzed using Student’s *t*-test with *p* < 0.05 considered significant.

### 2.8. RNA-Seq Analysis of High-Density Edited Cotton Lines

To investigate the impact of high-density chromosomal editing on the transcriptome, RNA-seq was performed on three independent upland cotton (*Gossypium hirsutum*) lines harboring edits at 16 target sites on chromosome D03. The Jin668 inbred line, subjected to the same tissue culture and regeneration process as the edited lines, was used as the wild-type control. Total RNA was extracted from leaf tissues, and RNA integrity was verified prior to library construction. Sequencing libraries were prepared following the Illumina protocol and subjected to high-throughput sequencing.

Raw sequencing data were first quality-checked using FastQC (v0.11.9). Low-quality reads and adapter sequences were trimmed using Trimmomatic (v0.32). Cleaned reads were then aligned to the T2T reference genome of Jin668 using HISAT2 (v2.2.1). The resulting SAM files were converted to BAM format and sorted and indexed using SAMtools (v1.9). Transcript assembly and quantification were performed using StringTie (v2.1.4), and gene-level expression counts were obtained using featureCounts (v2.0.0).

Differential expression analysis between the edited lines and the control was conducted using the DESeq2 (v1.42.0) package in R (v4.4.2). Significantly differentially expressed genes (DEGs) were identified based on an adjusted *p*-value threshold (FDR < 0.05). Functional enrichment of DEGs was carried out using Gene Ontology (GO) and KEGG pathway analyses to identify biological processes and pathways associated with centromere function. Expression patterns of centromere-related genes were visualized through heatmaps generated in R (v4.4.2).

### 2.9. DNA Extraction and PacBio HiFi Sequencing

Leaf samples were collected from three transgenic cotton lines, and total DNA was extracted using the CTAB method. The extracted DNA was further purified with the QIAGEN kit (Q13343, QIAGEN, Hilden, Germany) and quantified using both NanoDrop (Thermo Fisher Scientific, Wilmington, DE, USA) and Qubit (Thermo Fisher Scientific, Waltham, MA, USA). Following PacBio’s standard protocol, a SMRTbell library was constructed using the purified DNA. Sequencing was performed on the PacBio Sequel II platform in CCS mode. After sequencing, data quality was assessed using FastQC (v0.11.9). Chromosomal coverage was analyzed with samtools depth, and sequence alignment to the reference genome [3] was conducted using pbmm2 (v1.13.0). Structural variants were identified with pbsv, annotated using AnnotSV (v3.4.6), and visualized with IGV (v2.17.4) to interpret chromosomal structural variations.

## 3. Results

### 3.1. Construction of a High-Density CRISPR/Cas12a Multiplex Editing System in Cotton

**Figure 1:**
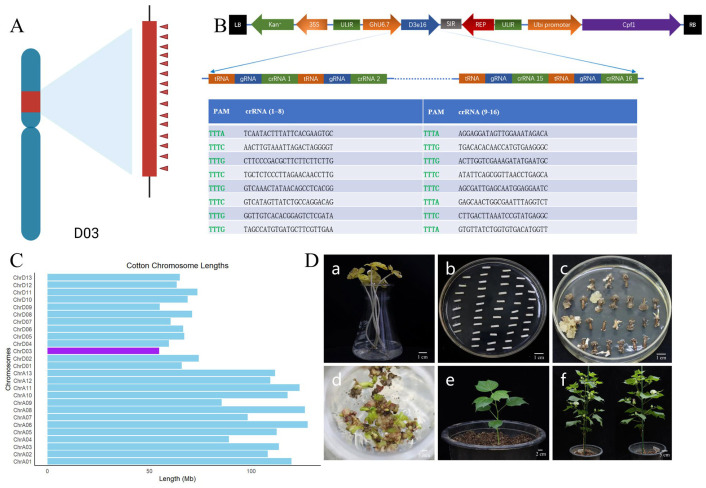
Design and construction of a CRISPR/Cas12a-based multiplex genome editing system targeting chromosome D03 in cotton. (**A**) Schematic diagram of 16 target sites within the 18.65–24.47 Mb region of chromosome D03, Detailed information for each target site is provided in Table S5. (**B**) Schematic diagram of the design of a 16-target editing vector based on pBeYDV-Cpf1. (**C**) Overview of chromosome lengths in *Gossypium hirsutum* cv. Jin668. (**D**) Agrobacterium tumefaciens-mediated transformation of cotton using Jin668 as the recipient genotype, showing key stages of tissue culture and regeneration, including (**a**) sterile etiolated seedlings, (**b**,**c**) hypocotyl explants cultured on kanamycin-containing modified MS medium, (**d**) callus induction and differentiation stage, and (**e**,**f**) regenerated plantlets grown under greenhouse conditions.

To enable high-density multiplex genome editing in cotton, a CRISPR/Cas12a-based system was developed by integrating a tRNA–crRNA polycistronic (PTC) processing strategy with a Bean yellow dwarf virus (BeYDV)-derived replicon. This design allows efficient processing and amplification of multiple crRNAs within plant cells, thereby enhancing multiplex editing capacity (Figure 1B).

Chromosome D03 of upland cotton (*Gossypium hirsutum* Jin668) was selected as the target due to its relatively small size (54.12 Mb) and the absence of known single-copy essential genes, making it a suitable model for high-density genome editing (Figure 1A). Based on the telomere-to-telomere (T2T) reference genome [3], a total of 16 target sites were designed within a conserved centromere-proximal region spanning 18.65–24.47 Mb on chromosome D03 (Figure 1A–C).

These target sequences were assembled into a single CRISPR/Cas12a construct (pBeYDV-LbCpf1-D03T16) using In-Fusion cloning. The resulting plasmid was introduced into cotton via Agrobacterium-mediated transformation (Figure 1D), generating transgenic lines for subsequent evaluation of multiplex editing efficiency and genomic effects.

### 3.2. Generation of High-Density Multiplex Edited Cotton Lines

Following transformation, regenerated plants were obtained under selective conditions and transferred to soil for further growth. Multiple independent transgenic lines were generated and screened by PCR for the presence of the CRISPR/Cas12a transgene.

A subset of positive lines was selected for subsequent analysis. These lines were advanced to the T1 generation, and four representative edited lines (designated as d3e1692, d3e1691, d3e16151, and d3e16152) were chosen for detailed characterization based on transformation success and plant viability. These selected lines were used for downstream analyses, including evaluation of editing efficiency, mutation profiling, phenotypic characterization, cytological examination, and transcriptomic analysis.

### 3.3. Editing Efficiency of High-Density Multiplex Genome Editing

To evaluate the efficiency of high-density multiplex genome editing, editing frequencies at individual target sites were quantified using barcode sequencing [20] and further validated by PacBio HiFi sequencing.

**Figure 2:**
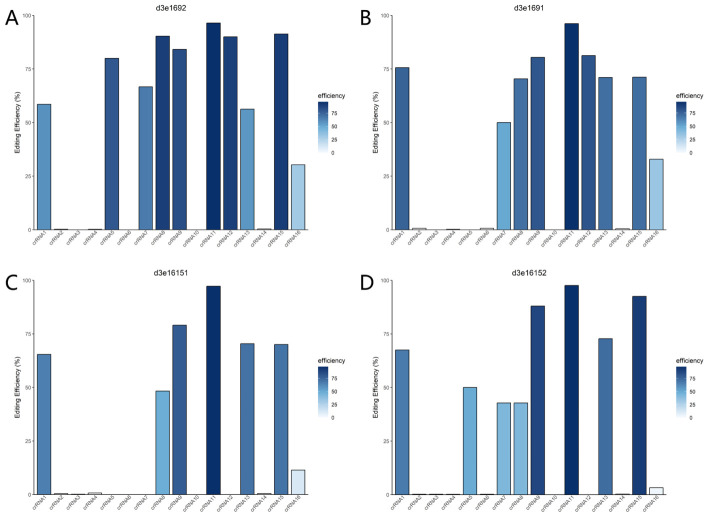
Editing efficiency across four transgenic lines carrying the D03T16 multiplex editing construct. (**A**–**D**) Editing efficiencies at individual target sites in four independent transgenic lines (d3e1692, d3e1691, d3e16151, and d3e16152), presented as bar charts.

Across the analyzed lines, multiple target loci exhibited high editing efficiencies (Table S3). Notably, in the D03T16-edited line d3e1692, up to 10 of the 16 target sites were edited within a single plant, demonstrating the feasibility of high-density multiplex genome editing in cotton (Figure 2A). Among the edited loci, nine exhibited editing efficiencies above 56%, with the highest efficiency reaching 96.46%. In contrast, a subset of target sites displayed relatively low or undetectable editing activity, indicating locus-dependent variability in editing efficiency. This variation may be attributed to differences in chromatin accessibility, sequence context, or crRNA processing efficiency.

To further evaluate the performance and consistency of the CRISPR/Cas12a multiplex editing system, editing efficiencies at individual target sites were compared across four independent transgenic lines harboring the same D03T16 construct (d3e1692, d3e1691, d3e16151, and d3e16152) (Figure 2A–D). Among the 16 target sites, simultaneous editing of up to 10 loci was detected in the d3e1692 line, whereas 9, 7, and 9 edited loci were identified in d3e1691, d3e16151, and d3e16152, respectively. Across all four lines, crRNA11 consistently exhibited the highest editing efficiency, ranging from 96.16% to 97.56%, while crRNA1, crRNA9, crRNA13, and crRNA15 also showed relatively high and stable editing frequencies across multiple lines. In contrast, crRNA16 displayed highly variable editing efficiencies, ranging from 3.22% to 32.87%. Notably, no detectable editing events were observed at crRNA2, crRNA3, crRNA4, crRNA10, or crRNA14 in any of the four transgenic lines. Overall, editing efficiencies across detectable targets ranged from 3.22% to 97.56%, indicating that the CRISPR/Cas12a system enables efficient and coordinated high-density multiplex genome editing in cotton, although editing activity varies substantially among individual target sites.

**Figure 3:**
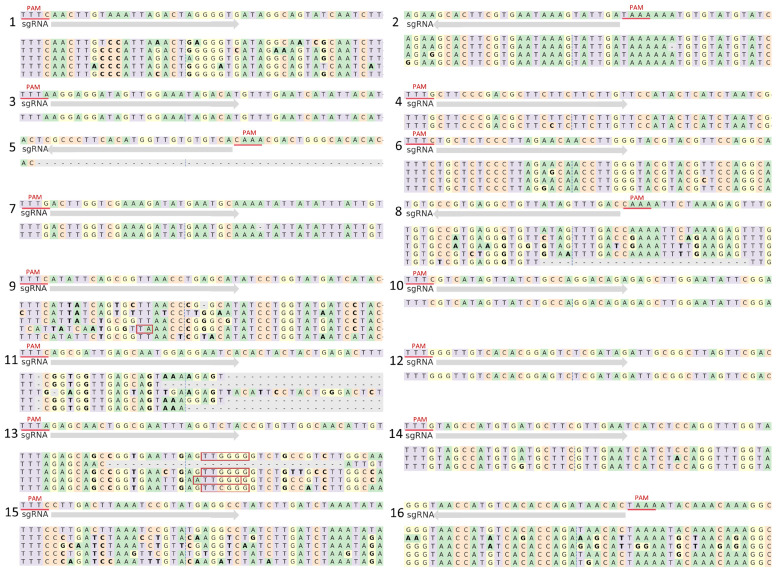
Editing patterns in the cotton line d3e16152. Panels 1–16 represent the editing patterns detected at each individual target site. Gray arrows indicate the orientations of the sgRNAs, while red underlines denote the protospacer adjacent motif (PAM) sites.

To further characterize the mutation profiles induced by Cas12a editing, representative editing patterns in line d3e16152 were analyzed using barcode amplicon sequencing (Table S4). Most mutations were distributed around the predicted Cas12a cleavage region approximately 18 bp downstream of the PAM site, including substitutions, deletions, and insertions, which is consistent with the characteristic cleavage pattern of Cas12a (Figure 3).

Overall, these results demonstrate that the developed CRISPR/Cas12a system enables efficient and high-density multiplex genome editing in cotton, while also highlighting the influence of target-specific factors on editing outcomes.

### 3.4. Mutation Profiling and Validation by HiFi Sequencing

To characterize the mutation patterns generated by high-density multiplex genome editing, targeted deep sequencing was performed using a barcode-based approach across the 16 edited loci. The results revealed diverse mutation types, predominantly consisting of small insertions and deletions (indels), which are characteristic of non-homologous end joining (NHEJ)-mediated DNA repair.

Across different target sites, mutation profiles showed considerable heterogeneity in both mutation frequency and indel size distribution. While some loci exhibited high frequencies of short deletions, others showed more complex mutation patterns, suggesting locus-dependent differences in DNA repair outcomes.

**Figure 4:**
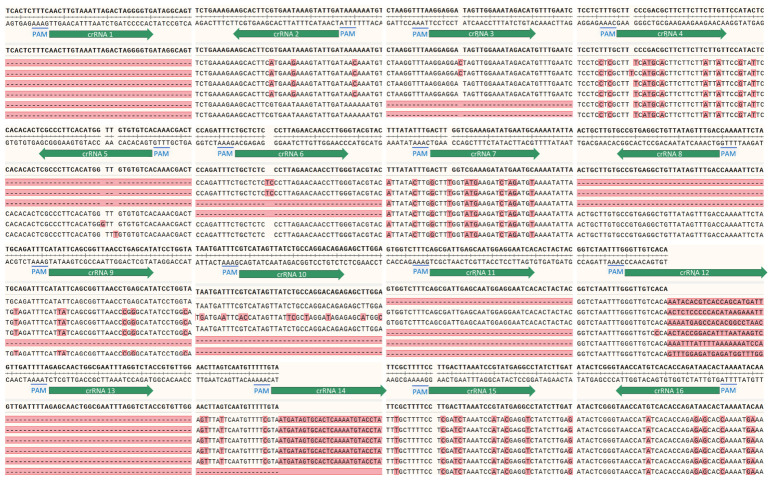
HiFi long-read sequencing confirms efficient and precise multiplex editing of cotton centromeric regions. Targets 1, 3, 4, 6, 7, 9, 10, 11, 12, 13, 14, and 15 are oriented in the forward direction, whereas targets 2, 5, 8, and 16 are oriented in the reverse direction. Green arrows indicate sgRNA orientations, and blue underlines mark the protospacer adjacent motif (PAM) sites.

To further validate these editing events at high resolution, PacBio HiFi long-read sequencing was employed. Representative HiFi reads were manually aligned using BLASTn (v2.14.1) and visualized with SnapGene (v7.2.1) software to qualitatively illustrate editing patterns and local sequence variations within edited regions. The HiFi data confirmed the presence of editing events at multiple target loci within individual plants, consistent with the barcode sequencing results (Figure 4). Importantly, the mutation patterns identified by HiFi sequencing were highly concordant with those obtained from barcode analysis, supporting the reliability and accuracy of the multiplex editing system.

Visualization of HiFi reads using IGV (v2.16.2) further revealed localized clustering of breakpoint signals and rearranged junction patterns within edited regions, providing additional evidence for targeted editing-induced DNA modifications. These observations indicate that high-density CRISPR/Cas12a-mediated editing can generate diverse and site-specific mutation outcomes without causing large-scale chromosomal rearrangements.

Together, these results demonstrate that the developed system enables efficient induction of mutations across multiple genomic loci and that HiFi sequencing provides a robust approach for comprehensive validation of multiplex genome editing events.

### 3.5. Phenotypic Effects of High-Density Multiplex Genome Editing

To assess the impact of high-density multiplex genome editing on plant development, transgenic cotton lines carrying the CRISPR/Cas12a construct targeting 16 loci on chromosome D03 were grown under controlled greenhouse conditions, with wild-type Jin668 plants serving as the control.

**Figure 5:**
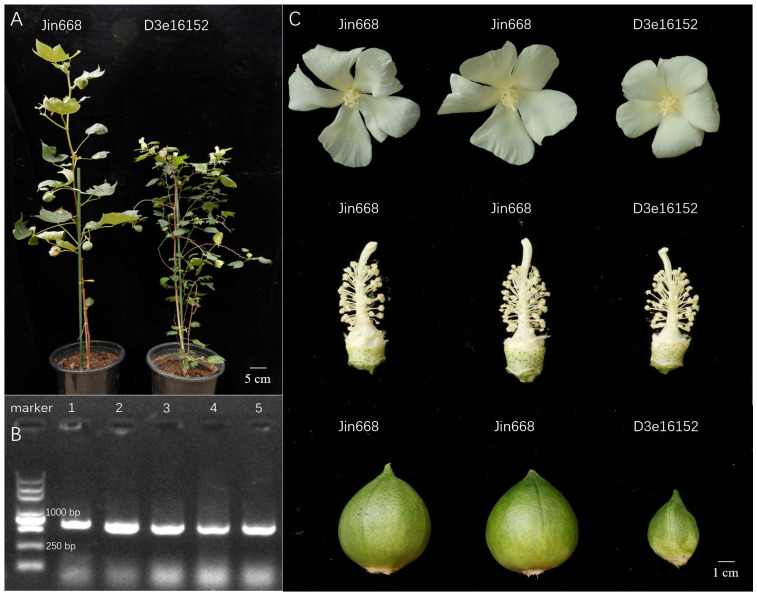
Phenotypic characterization of the D03 chromosome multiplex-edited cotton line d3e16152. (**A**) Morphological comparison between the 16-target edited line d3e16152 and the wild-type control Jin668. (**B**) PCR-based verification of the edited line d3e16152. (**C**) Morphological comparison of floral organs (flowers, pistils, and buds) between d3e16152 and the wild-type Jin668.

Compared with the wild type, all edited lines exhibited varying degrees of growth inhibition (Figure 5A–C). The most prominent phenotypic alterations included reduced plant vigor, slower growth rates, and overall stunted development. Notably, substantial variation in phenotypic severity was observed among independent edited lines. Among them, the d3e16152 line displayed the most pronounced growth defects, whereas other lines showed moderate or relatively mild phenotypic changes.

Given that the targeted loci are located within non-coding regions of chromosome D03, the observed developmental abnormalities are unlikely to result from direct disruption of protein-coding genes. Instead, these phenotypes may be associated with perturbations of regulatory elements or broader chromosomal effects induced by high-density genome editing. In particular, the simultaneous induction of multiple DNA double-strand breaks may compromise genome integrity, thereby affecting normal cellular processes and plant growth.

Collectively, these results indicate that high-density multiplex genome editing can lead to measurable developmental defects in cotton and suggest that editing density may be an important factor influencing phenotypic outcomes.

### 3.6. Cytological Defects Induced by High-Density Multiplex Genome Editing

To further investigate the cellular basis underlying the observed growth defects, cytological analyses were performed using root tip cells and anthers from edited and wild-type plants. Microscopic examination revealed pronounced abnormalities in both mitotic and meiotic cell division in high-density edited lines.

**Figure 6:**
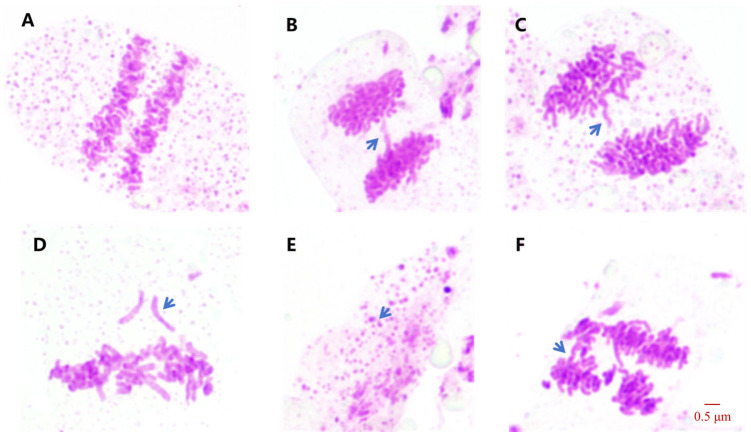
Cytological analysis of mitosis in root apical meristem cells of the 16-target multiplex-edited cotton line d3e16152. (**A**) Wild-type control. (**B**) Chromosome bridge. (**C**) Chromosome lagging. (**D**) Chromosome fragments. (**E**) Micronucleus formation. (**F**) Unequal chromosome segregation. Arrows in the diagram indicate regions associated with chromosome abnormalities.

In mitotic cells of the edited lines, multiple types of chromosomal segregation defects were observed (Figure 6A–F). These included chromosome bridges during anaphase (Figure 6B), lagging chromosomes (Figure 6C), and unequal chromosome segregation (Figure 6F), all of which indicate impaired chromosome stability and defective mitotic progression. In contrast, such abnormalities were rarely detected in wild-type plants (Figure 6A).

**Figure 7:**
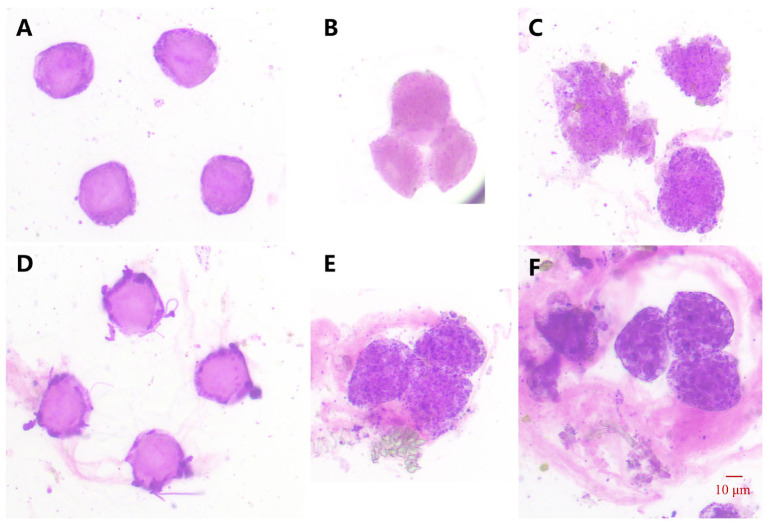
Abnormal tetrad formation in pollen mother cells of the 16-target multiplex-edited cotton line d3e16152. (**A**) Normal tetrad formation in the wild-type control. (**B**–**F**) Representative abnormal meiotic products observed in the edited line, including triad formation and unequal meiotic division.

Consistent with the mitotic defects, meiotic analysis of the edited lines also revealed abnormal meiotic products. Instead of normal tetrad formation, irregular meiotic products such as triads and unequal division patterns were frequently observed in the edited lines, indicating abnormal meiotic progression and chromosome segregation defects (Figure 7A–F).

Notably, the severity of cytological abnormalities was consistent with the observed phenotypic differences among edited lines. The d3e16152 line, which exhibited the most severe growth defects, also showed the highest frequency of cytological abnormalities, suggesting a correlation between editing-induced genomic perturbations and cellular dysfunction.

These results provide direct cytological evidence that high-density multiplex genome editing can compromise chromosome stability and interfere with both mitotic and meiotic processes. The widespread occurrence of chromosomal abnormalities suggests that the simultaneous induction of multiple DNA double-strand breaks may exceed the cellular DNA repair capacity, thereby leading to defects in chromosome segregation and genome maintenance.

### 3.7. Transcriptomic Changes Associated with High-Density Multiplex Genome Editing

To further investigate the molecular basis underlying the observed cytological abnormalities and growth defects, transcriptome profiling was performed using RNA-seq on three independent edited cotton lines, with wild-type Jin668 plants serving as the control.

**Figure 8:**
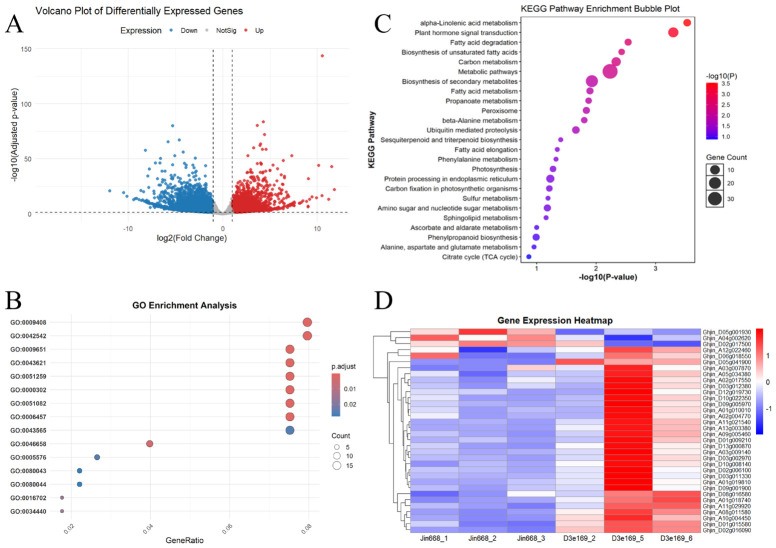
Transcriptome analysis of the effects of high-density multiplex editing on chromosome D03 in cotton. (**A**) Volcano plot showing genome-wide differential gene expression between D03 16-target multiplex-edited plants and the wild-type Jin668. (**B**) Gene Ontology (GO) enrichment analysis of DEGs associated with chromosome segregation, centromere assembly, and cell cycle regulation. (**C**) KEGG pathway enrichment analysis of differentially expressed genes (DEGs). (**D**) Heatmap of representative DEGs related to chromosome dynamics and cell division.

Comparative transcriptomic analysis revealed widespread changes in gene expression in the edited lines relative to the wild type. A substantial number of differentially expressed genes (DEGs) were identified, indicating that high-density multiplex genome editing exerts a broad impact on transcriptional regulation (Figure 8A).

Gene functional annotation and enrichment analysis showed that the identified transcriptional changes were associated with biological processes related to cell division, including kinetochore assembly, spindle organization, and cell cycle regulation (Figure 8B–D). Notably, several genes essential for chromosome segregation and mitotic progression, such as *NDC80* and *CENPE*, were differentially expressed in the edited lines (Table 1).

The dysregulation of these genes provides a plausible molecular explanation for the cytological defects observed in mitosis and meiosis. Given the critical roles of kinetochore-associated proteins and spindle assembly factors in ensuring accurate chromosome segregation, their altered expression may lead to chromosome missegregation, resulting in phenotypes such as lagging chromosomes, chromosome bridges, and abnormal meiotic products.

In addition, the global transcriptional perturbations observed in edited lines suggest that high-density genome editing may influence not only local genomic regions but also broader regulatory networks. This effect may be mediated through DNA damage responses or alterations in higher-order chromatin organization induced by multiple simultaneous double-strand breaks.

Collectively, these results indicate that high-density multiplex genome editing can lead to extensive transcriptional reprogramming, which in turn contributes to defects in chromosome segregation and plant development.

### 3.8. Genome Stability Analysis in High-Density Edited Cotton Lines

To assess the impact of high-density multiplex genome editing on genome integrity, PacBio HiFi long-read sequencing data were analyzed to detect potential large-scale structural variations in edited cotton lines.

Consistent with the targeted editing design, sequence alterations were predominantly confined to the intended target regions on chromosome D03. Local sequence variations, including insertions and deletions, were detected at multiple target loci, confirming efficient editing activity. However, no evidence of large-scale chromosomal rearrangements, such as translocations, inversions, or extensive deletions, was observed at the genome-wide level.

Despite the absence of detectable large structural variations, the edited lines exhibited pronounced cytological abnormalities and developmental defects, as described above. This apparent discrepancy suggests that high-density multiplex genome editing may impair genome function without necessarily inducing large-scale structural changes.

One possible explanation is that the simultaneous induction of multiple DNA double-strand breaks leads to localized genomic perturbations and cumulative DNA damage responses, which may disrupt chromosome behavior during cell division. In addition, these perturbations may alter higher-order chromatin organization, including chromatin looping, long-range regulatory interactions, and three-dimensional genome architecture. Such changes could interfere with gene regulatory networks and spatial genome organization, thereby contributing to transcriptional dysregulation and compromised genome stability.

Taken together, these results indicate that high-density CRISPR/Cas12a-mediated genome editing can preserve overall chromosomal structure while still exerting significant effects on genome stability, chromatin organization, and cellular function.

## 4. Discussion

In this study, we established a high-density multiplex genome editing system in cotton using CRISPR/Cas12a, enabling the simultaneous targeting of up to 16 genomic loci. By integrating a tRNA–crRNA polycistronic processing strategy with a BeYDV-derived replicon, we achieved efficient and coordinated expression of multiple crRNAs in a complex polyploid genome. Compared with previous plant studies, this system represents a substantial expansion in multiplex editing capacity and demonstrates the scalability of Cas12a-based platforms for large-scale genome engineering [21,22].

A key advantage of the Cas12a system lies in its intrinsic ability to process crRNA arrays, which enables compact and efficient multiplex editing without the need for additional RNA processing components [9,23]. In this study, the combination of the PTC system and viral replicon markedly enhanced crRNA abundance and editing efficiency, allowing simultaneous editing of up to 10 target sites within a single plant. This highlights the scalability of Cas12a-based multiplex editing and its suitability for large-scale genome engineering, particularly in polyploid species characterized by high gene redundancy. Compared with Cas9-based systems, which typically require multiple independent sgRNA expression cassettes, the Cas12a system offers a more streamlined and efficient strategy for multiplex genome targeting [24].

Beyond technical advancement, our results reveal important biological consequences of high-density genome editing. Although HiFi long-read sequencing did not detect large-scale chromosomal rearrangements, edited plants exhibited pronounced developmental defects and cytological abnormalities, including chromosome bridges, lagging chromosomes, and irregular meiotic products. These phenotypes are hallmarks of genome instability and suggest that the simultaneous induction of multiple DNA double-strand breaks (DSBs) imposes a substantial burden on cellular DNA repair systems. We propose that high-density multiplex editing may generate a level of DNA damage that exceeds the repair capacity of the cell, leading to defects in chromosome segregation and cell division [25,26,27].

At the molecular level, transcriptome profiling revealed transcriptional reprogramming associated with biological processes related to kinetochore assembly, spindle organization, and cell cycle progression, including genes such as *NDC80* and *CENPE* [28]. Rather than representing direct primary targets of genome editing, these transcriptional changes likely reflect downstream cellular and adaptive responses to genome perturbation, DNA damage signaling, altered chromatin organization, and disrupted cellular homeostasis induced by high-density multiplex genome editing. Because bulk RNA-seq captures averaged transcriptional outputs across heterogeneous cell populations, the identified differentially expressed genes (DEGs) should be interpreted as components of broader compensatory and regulatory responses rather than direct causal determinants of the observed phenotypes. Notably, the absence of large-scale structural variations suggests that the observed genome instability is not primarily driven by gross chromosomal rearrangements, but may instead arise from cumulative local perturbations in chromatin structure and genome function.

It is important to note that gene expression is inherently cell type– and tissue-specific in plants, and bulk transcriptomic profiling may mask cell type–resolved regulatory dynamics. In this study, RNA-seq was performed using whole-leaf tissue, and thus the differentially expressed genes (DEGs) identified represent averaged transcriptional responses across multiple cell types rather than cell type–specific expression changes. Consequently, spatial or cell type–specific regulatory effects that may contribute to the observed phenotypic variations cannot be resolved in the present analysis. Future studies using single-cell RNA sequencing or spatial transcriptomics will be valuable to further dissect the cellular basis of transcriptional responses induced by high-density genome editing.

An additional layer of complexity may arise from the impact of high-density editing on higher-order genome organization. Increasing evidence from chromatin conformation studies indicates that genome function is tightly linked to three-dimensional chromatin architecture, including chromatin loops and topologically associating domains (TADs) [29,30]. The simultaneous introduction of multiple DSBs within a confined chromosomal region may perturb local chromatin interactions, thereby altering regulatory landscapes and gene expression patterns. Although direct evidence remains to be established, this hypothesis provides a plausible framework linking multiplex genome editing to global transcriptional and cellular phenotypes.

Collectively, our findings point to the existence of a potential “editing density threshold” in multiplex genome editing. While increasing the number of target sites enhances the power of functional genomics and enables coordinated manipulation of multiple loci, excessive editing density may compromise genome integrity and developmental stability. Although direct evidence for such trade-offs in plant multiplex editing remains limited, it is well established that excessive induction of DNA double-strand breaks can exceed cellular repair capacity, thereby compromising genome stability [8,31]. This relationship underscores the need to balance editing efficiency with biological constraints, particularly in complex polyploid genomes.

In conclusion, this study demonstrates the feasibility of high-density multiplex genome editing in cotton and provides new conceptual insights into its biological constraints. The CRISPR/Cas12a-based platform developed here represents a versatile tool for large-scale genome engineering in polyploid crops. Importantly, our findings highlight the impact of editing density on genome stability and cellular function, underscoring the need to balance editing efficiency with genome integrity in multiplex genome editing applications.

## Figures and Tables

**Table 1:** Chromosomal segregation-related differentially expressed genes.

Gene_ID	Description		Gene Functional Classification
Ghjin_A01g010010	Inner centromere protein ARK-binding domain-containing protein		Cenp-related protein
Ghjin_D01g009210	Inner centromere protein ARK-binding domain-containing protein		
Ghjin_D10g008140	Centromere protein C		
Ghjin_A03g007870	Structural maintenance of chromosomes protein		Chromosome structure-related proteins
Ghjin_D03g012380	Structural maintenance of chromosomes protein		
Ghjin_D09g001900	Structural maintenance of chromosomes protein		
Ghjin_D13g000870	Mini-chromosome maintenance complex-binding protein		
Ghjin_A08g011580	Chromosome transmission fidelity protein 8		
Ghjin_A02g004770	Minichromosome loss protein Mcl1 middle region domain-containing protein		
Ghjin_D02g006100	Minichromosome loss protein Mcl1 middle region domain-containing protein		
Ghjin_A01g019810	Kinetochore protein NDC80		Kinetochore protein
Ghjin_A01g018740	Meiosis-specific protein ASY3-like coiled-coil domain-containing protein		Meiosis-specific protein
Ghjin_A04g002620	Spindle pole body component protein		Spindle pole body protein
Ghjin_A02g017550	Microtubule-associated protein RP/EB family member 1C		Microtubule-associated protein
Ghjin_A09g005460	65 kDa microtubule-associated protein 3-like		
Ghjin_A11g029920	Microtubule-binding protein TANGLED		
Ghjin_D01g015580	187 kDa microtubule-associated protein AIR9		
Ghjin_D02g016090	65 kDa microtubule-associated protein 6		
Ghjin_D02g017500	65 kDa microtubule-associated protein 8		
Ghjin_D03g002970	Microtubule-associated protein RP/EB family member 1C		
Ghjin_D05g041900	65 kDa microtubule-associated protein 1		
Ghjin_D06g018550	Microtubule-associated protein 70-5-like		
Ghjin_D08g016580	65 kDa microtubule-associated protein 4-like		
Ghjin_D09g005970	65 kDa microtubule-associated protein 3		
Ghjin_D10g022350	65 kDa microtubule-associated protein 5		
Ghjin_D12g019730	Microtubule-associated protein futsch-like isoform X2		
Ghjin_A03g009140	Replication protein A subunit		Other related proteins
Ghjin_A05g034380	DNA polymerase alpha subunit B		
Ghjin_A10g004450	DNA replication licensing factor MCM3		
Ghjin_A11g021540	DNA polymerase		
Ghjin_A12g022460	Ribonuclease H2 subunit B		
Ghjin_A13g003380	DNA replication licensing factor MCM4		
Ghjin_D03g011330	Replication protein A subunit		
Ghjin_D05g001930	CST complex subunit CTC1		

## Data Availability

The raw barcode amplicon sequencing data, RNA-seq data, and PacBio HiFi sequencing data generated in this study have been deposited in the NCBI Sequence Read Archive (SRA) database under BioProject accession numbers PRJNA1468915, PRJNA1465025, and PRJNA1465669, respectively. These datasets will be publicly available upon publication.

## Supplementary Materials

The following supporting information can be downloaded at: https://www.mdpi.com/article/10.3390/genes17060622/s1, Table S1. PCR verification primer sequences; Table S2. Primer sequences used for barcode-based detection of multiplex-edited target sites; Table S3. D03 Chromosome 16 Target Editing Efficiency Detection. Table S4. Statistics on d3e1615 barcode sequencing reads and editing efficiency. Table S5. shows the detailed information of each target site.
