# Supplementary material for: High-Density CRISPR/Cas12a-Mediated Multiplex Genome Editing Reveals Genome Instability in Allotetraploid Cotton
Source: Genes (Basel). 2026 May 29;17(6):622. doi: 10.3390/genes17060622 (PMC13298417; doi:10.3390/genes17060622)
Supplement: Supplementary file 1 [file genes-17-00622-s001.zip › genes-4339280-supplementary.pdf]

Table S1. PCR verification primer sequences

Name	Sequence (5'-3')
Cpf1-p-F	GCGACAATTCTCCGCTATG
Cpf1-p-R	TGTGGCAGTCGTTGAGGTTA
cpf1-2p-F	GGCTTCGTGAACCTCCTGAA
cpf1-2p-R	GTCGAGCTTCTCATCCTCCG

Table S2. Primer sequences used for barcode-based detection of multiplex-edited target sites

Target site	Target sequence (5'-3')
bD3-402-F	GACgtggacGAACACACTTGACTTCTAAGGTCTT
bD3-402-R	GACagctcaATAGAATCAAGGTGACTTTCGTTAC
bD3-805-F	GACgtggacCCTCTTTATCATCCATATATTCGTC
bD3-805-R	GACagctcaGTTGGCAGGAACTCAATAAGAC
bD3-1207-F	GACgtggacCGACCCACACGCTGAAA
bD3-1207-R	GACagctcaAGCTGCATGTGTAGTTGGTTAA
bD3-1610-F	GACgtggacGATAATAGCTGCTTCTGCTGCT
bD3-1610-R	GACagctcaTAGAGACAATACACAGTCACCCAA
bD3-2013-F	GACgtggacAATCTCATAGTCATAAAGTTAATTCGA
bD3-2013-R	GACagctcaATTTGAGTAGACGTTGCATGTTT
bD3-2415-F	GACgtggacCCTCTATCTCTAGTCAGTAGGCTTAC
bD3-2415-R	GACagctcaCACAATCCCCTCAACCATC
bD3-2817-F	GACgtggacCTCGACTTCAAATGAGCAAAGT
bD3-2817-R	GACagctcaGCCAGATCCTATCTCACAAGG
bD3-3220-F	GACgtggacGCCTAAATGGCGAGTCGT
bD3-3220-R	GACagctcaAATGCCAGTCACACCGTATC
bD3-3620-F	GACgtggacCACGACGGTGCCAACG
bD3-3620-R	GACagctcaTTTATACAGGGAGAGTTTAGAGAGTTC
bD3-4027-F	GACgtggacTGGCTCGGTAATGTGGTCT
bD3-4027-R	GACagctcaCTGAACCCTAGCCTCATTACTC
bD3-4100-F	GACgtggacGAATACGATATGCTCGGACATAC
bD3-4100-R	GACagctcaTATGAGTGGGTATGTATATGGGTAG

bD3-4830-F	GACgtggacTCATATTCACCAATTGGCTCA
bD3-4830-R	GACagctcaATAGCAGATGTAGAATCAACCACTT
bD3-5323-F	GACgtggacGGAACTTACGTCGACAAACAA
bD3-5323-R	GACagctcaATGCTTAGATTTCAATTTATGAGC
bD3-5432-F	GACgtggacCCTTCTAATGGGTAAACACCAA
bD3-5432-R	GACagctcaGTATGGGTTTGCACTTCGG
bD3-qian500-F	GACgtggacATGTTGTATTCATGCTTGTTCG
bD3-qian500-R	GACagctcaGTCTGGTATTGGTACATTGATCC
bD3-hou500-F	GACgtggacCCAGAGACTAACCGATAAGGAGT
bD3-hou500-R	GACagctcaCGATTTGGGACACCAAGAG
b1D3-402-F	GACgacatgGAACACACTTGACTTCTAAGGTCTT
b1D3-402-R	GACagatgtATAGAATCAAGGTGACTTTCGTTAC
b1D3-805-F	GACgacatgCCTCTTTATCATCCATATATTCGTC
b1D3-805-R	GACagatgtGTTGGCAGGAACTCAATAAGAC
b1D3-1207-F	GACgacatgCGACCCACACGCTGAAA
b1D3-1207-R	GACagatgtAGCTGCATGTGTAGTTGGTTAA
b1D3-1610-F	GACgacatgGATAATAGCTGCTTCTGCTGCT
b1D3-1610-R	GACagatgtTAGAGACAATACACAGTCACCCAA
b1D3-2013-F	GACgacatgAATCTCATAGTCATAAAGTTAATTCGA
b1D3-2013-R	GACagatgtATTTGAGTAGACGTTGCATGTTT
b1D3-2415-F	GACgacatgCCTCTATCTCTAGTCAGTAGGCTTAC
b1D3-2415-R	GACagatgtCACAATCCCCTCAACCATC
b1D3-2817-F	GACgacatgCTCGACTTCAAATGAGCAAAGT
b1D3-2817-R	GACagatgtGCCAGATCCTATCTCACAAGG
b1D3-3220-F	GACgacatgGCCTAAATGGCGAGTCGT
b1D3-3220-R	GACagatgtAATGCCAGTCACACCGTATC
b1D3-3620-F	GACgacatgCACGACGGTGCCAACG
b1D3-3620-R	GACagatgtTTTATACAGGGAGAGTTTAGAGAGTTC
b1D3-4027-F	GACgacatgTGGCTCGGTAATGTGGTCT
b1D3-4027-R	GACagatgtCTGAACCCTAGCCTCATTACTC
b1D3-4100-F	GACgacatgGAATACGATATGCTCGGACATAC
b1D3-4100-R	GACagatgtTATGAGTGGGTATGTATATGGGTAG
b1D3-4830-F	GACgacatgTCATATTCACCAATTGGCTCA
b1D3-4830-R	GACagatgtATAGCAGATGTAGAATCAACCACTT

b1D3-5323-F	GACgacatgGGAACTTACGTCGACAAACAA
b1D3-5323-R	GACagatgtATGCTTAGATTTCAATTTATGAGC
b1D3-5432-F	GACgacatgCCTTCTAATGGGTAAACACCAA
b1D3-5432-R	GACagatgtGTATGGGTTTGCACTTCGG
b1D3-qian500-F	GACgacatgATGTTGTATTCATGCTTGTTCG
b1D3-qian500-R	GACagatgtGTCTGGTATTGGTACATTGATCC
b1D3-hou500-F	GACgacatgCCAGAGACTAACCGATAAGGAGT
b1D3-hou500-R	GACagatgtCGATTTGGGACACCAAGAG
b2D3-402-F	GACgtcacgGAACACACTTGACTTCTAAGGTCTT
b2D3-402-R	GACatggtcATAGAATCAAGGTGACTTTCGTTAC
b2D3-805-F	GACgtcacgCCTCTTTATCATCCATATATTCGTC
b2D3-805-R	GACatggtcGTTGGCAGGAACTCAATAAGAC
b2D3-1207-F	GACgtcacgCGACCCACACGCTGAAA
b2D3-1207-R	GACatggtcAGCTGCATGTGTAGTTGGTTAA
b2D3-1610-F	GACgtcacgGATAATAGCTGCTTCTGCTGCT
b2D3-1610-R	GACatggtcTAGAGACAATACACAGTCACCCAA
b2D3-2013-F	GACgtcacgAATCTCATAGTCATAAAGTTAATTCGA
b2D3-2013-R	GACatggtcATTTGAGTAGACGTTGCATGTTT
b2D3-2415-F	GACgtcacgCCTCTATCTCTAGTCAGTAGGCTTAC
b2D3-2415-R	GACatggtcCACAATCCCCTCAACCATC
b2D3-2817-F	GACgtcacgCTCGACTTCAAATGAGCAAAGT
b2D3-2817-R	GACatggtcGCCAGATCCTATCTCACAAGG
b2D3-3220-F	GACgtcacgGCCTAAATGGCGAGTCGT
b2D3-3220-R	GACatggtcAATGCCAGTCACACCGTATC
b2D3-3620-F	GACgtcacgCACGACGGTGCCAACG
b2D3-3620-R	GACatggtcTTTATACAGGGAGAGTTTAGAGAGTTC
b2D3-4027-F	GACgtcacgTGGCTCGGTAATGTGGTCT
b2D3-4027-R	GACatggtcCTGAACCCTAGCCTCATTACTC
b2D3-4100-F	GACgtcacgGAATACGATATGCTCGGACATAC
b2D3-4100-R	GACatggtcTATGAGTGGGTATGTATATGGGTAG
b2D3-4830-F	GACgtcacgTCATATTCACCAATTGGCTCA
b2D3-4830-R	GACatggtcATAGCAGATGTAGAATCAACCACTT
b2D3-5323-F	GACgtcacgGGAACTTACGTCGACAAACAA
b2D3-5323-R	GACatggtcATGCTTAGATTTCAATTTATGAGC

b2D3-5432-F	GACgtcacgCCTTCTAATGGGTAAACACCAA
b2D3-5432-R	GACatggtcGTATGGGTTTGCACTTCGG
b2D3-qian500-F	GACgtcacgATGTTGTATTCATGCTTGTTCG
b2D3-qian500-R	GACatggtcGTCTGGTATTGGTACATTGATCC
b2D3-hou500-F	GACgtcacgCCAGAGACTAACCGATAAGGAGT
b2D3-hou500-R	GACatggtcCGATTTGGGACACCAAGAG
b3D3-402-F	GACgtcgctGAACACACTTGACTTCTAAGGTCTT
b3D3-402-R	GACatgctgATAGAATCAAGGTGACTTTCGTTAC
b3D3-805-F	GACgtcgctCCTCTTTATCATCCATATATTCGTC
b3D3-805-R	GACatgctgGTTGGCAGGAACTCAATAAGAC
b3D3-1207-F	GACgtcgctCGACCCACACGCTGAAA
b3D3-1207-R	GACatgctgAGCTGCATGTGTAGTTGGTTAA
b3D3-1610-F	GACgtcgctGATAATAGCTGCTTCTGCTGCT
b3D3-1610-R	GACatgctgTAGAGACAATACACAGTCACCCAA
b3D3-2013-F	GACgtcgctAATCTCATAGTCATAAAGTTAATTCGA
b3D3-2013-R	GACatgctgATTTGAGTAGACGTTGCATGTTT
b3D3-2415-F	GACgtcgctCCTCTATCTCTAGTCAGTAGGCTTAC
b3D3-2415-R	GACatgctgCACAATCCCCTCAACCATC
b3D3-2817-F	GACgtcgctCTCGACTTCAAATGAGCAAAGT
b3D3-2817-R	GACatgctgGCCAGATCCTATCTCACAAGG
b3D3-3220-F	GACgtcgctGCCTAAATGGCGAGTCGT
b3D3-3220-R	GACatgctgAATGCCAGTCACACCGTATC
b3D3-3620-F	GACgtcgctCACGACGGTGCCAACG
b3D3-3620-R	GACatgctgTTTATACAGGGAGAGTTTAGAGAGTTC
b3D3-4027-F	GACgtcgctTGGCTCGGTAATGTGGTCT
b3D3-4027-R	GACatgctgCTGAACCCTAGCCTCATTACTC
b3D3-4100-F	GACgtcgctGAATACGATATGCTCGGACATAC
b3D3-4100-R	GACatgctgTATGAGTGGGTATGTATATGGGTAG
b3D3-4830-F	GACgtcgctTCATATTCACCAATTGGCTCA
b3D3-4830-R	GACatgctgATAGCAGATGTAGAATCAACCACTT
b3D3-5323-F	GACgtcgctGGAACTTACGTCGACAAACAA
b3D3-5323-R	GACatgctgATGCTTAGATTTCAATTTATGAGC
b3D3-5432-F	GACgtcgctCCTTCTAATGGGTAAACACCAA
b3D3-5432-R	GACatgctgGTATGGGTTTGCACTTCGG

b3D3-qian500-F	GACgtcgctATGTTGTATTCATGCTTGTTCG
b3D3-qian500-R	GACatgctgGTCTGGTATTGGTACATTGATCC
b3D3-hou500-F	GACgtcgctCCAGAGACTAACCGATAAGGAGT
b3D3-hou500-R	GACatgctgCGATTTGGGACACCAAGAG
b4D3-402-F	GACgtcgtaGAACACACTTGACTTCTAAGGTCTT
b4D3-402-R	GACagcacgATAGAATCAAGGTGACTTTCGTTAC
b4D3-805-F	GACgtcgtaCCTCTTTATCATCCATATATTCGTC
b4D3-805-R	GACagcacgGTTGGCAGGAACTCAATAAGAC
b4D3-1207-F	GACgtcgtaCGACCCACACGCTGAAA
b4D3-1207-R	GACagcacgAGCTGCATGTGTAGTTGGTTAA
b4D3-1610-F	GACgtcgtaGATAATAGCTGCTTCTGCTGCT
b4D3-1610-R	GACagcacgTAGAGACAATACACAGTCACCCAA
b4D3-2013-F	GACgtcgtaAATCTCATAGTCATAAAGTTAATTCGA
b4D3-2013-R	GACagcacgATTTGAGTAGACGTTGCATGTTT
b4D3-2415-F	GACgtcgtaCCTCTATCTCTAGTCAGTAGGCTTAC
b4D3-2415-R	GACagcacgCACAATCCCCTCAACCATC
b4D3-2817-F	GACgtcgtaCTCGACTTCAAATGAGCAAAGT
b4D3-2817-R	GACagcacgGCCAGATCCTATCTCACAAGG
b4D3-3220-F	GACgtcgtaGCCTAAATGGCGAGTCGT
b4D3-3220-R	GACagcacgAATGCCAGTCACACCGTATC
b4D3-3620-F	GACgtcgtaCACGACGGTGCCAACG
b4D3-3620-R	GACagcacgTTTATACAGGGAGAGTTTAGAGAGTTC
b4D3-4027-F	GACgtcgtaTGGCTCGGTAATGTGGTCT
b4D3-4027-R	GACagcacgCTGAACCCTAGCCTCATTACTC
b4D3-4100-F	GACgtcgtaGAATACGATATGCTCGGACATAC
b4D3-4100-R	GACagcacgTATGAGTGGGTATGTATATGGGTAG
b4D3-4830-F	GACgtcgtaTCATATTCACCAATTGGCTCA
b4D3-4830-R	GACagcacgATAGCAGATGTAGAATCAACCACTT
b4D3-5323-F	GACgtcgtaGGAACTTACGTCGACAAACAA
b4D3-5323-R	GACagcacgATGCTTAGATTTCAATTTATGAGC
b4D3-5432-F	GACgtcgtaCCTTCTAATGGGTAAACACCAA
b4D3-5432-R	GACagcacgGTATGGGTTTGCACTTCGG
b4D3-qian500-F	GACgtcgtaATGTTGTATTCATGCTTGTTCG
b4D3-qian500-R	GACagcacgGTCTGGTATTGGTACATTGATCC

b4D3-hou500-F	GACgtcgtaCCAGAGACTAACCGATAAGGAGT
b4D3-hou500-R	GACagcacgCGATTTGGGACACCAAGAG

Table S3. D03 Chromosome 16 Target Editing Efficiency Detection

Target	D3-e-16 9（2）	D3-e-16 15（2）	D3-e-16 9	D3-e-16 15
crRNA1	58.54	67.52	75.63	65.42
crRNA2	0.24	0.22	0.64	0.47
crRNA3	NA	0.22	NA	0.2
crRNA4	0.22	0.2	0.21	0.75
crRNA5	80	50	NA	NA
crRNA6	NA	0.23	0.65	NA
crRNA7	66.67	42.8	50	NA
crRNA8	90.3	42.8	70.4	48.31
crRNA9	84.15	88	80.41	79.05
crRNA10	NA	NA	50	NA
crRNA11	96.46	97.56	96.16	97.24
crRNA12	90	NA	81.25	NA
crRNA13	56.24	72.75	71.04	70.39
crRNA14	0.34	0.32	0.49	0.52
crRNA15	91.34	92.51	71.21	70.03
crRNA16	30.35	3.22	32.87	11.42

Table S4. Statistics on d3e1615 barcode sequencing reads and editing efficiency

Target	Reads aligned	Modified reads	Editing efficiency(%)
crRNA1	156555	105705	67.52
crRNA2	25400	55	0.22
crRNA3	72448	159	0.22
crRNA4	196229	392	0.2
crRNA5	6021	3010	50
crRNA6	159192	366	0.23
crRNA7	31388	13434	42.8
crRNA8	124890	53452	42.8
crRNA9	24504	21563	88
crRNA10	5587	0	0
crRNA11	40898	39900	97.56
crRNA12	2091	0	0
crRNA13	1730	1258	72.75
crRNA14	150499	481	0.32
crRNA15	103713	95944	92.51
crRNA16	11727	377	3.22

Table S5. shows the detailed information of each target site.

target	Start position (bp)	End position (bp)	crRNA	PAM	primer-F	primer-R	amplicon sizes
crRNA1	19049539	19049561	AACTTGTAAATTAGACTAGGGGT	TTTC	CCTCTTTATCATCCATATATTCGTC	GTTGGCAGGAACTCAATAAGAC	271
crRNA2	18647084	18647106	TCAATACTTTATTCACGAAGTGC	TTTA	GAACACACTTGACTTCTAAGGTCTT	ATAGAATCAAGGTGACTTTCGTTAC	289
crRNA3	22038810	22038832	AGGAGGATAGTTGGAAATAGACA	TTTA	CACGACGGTGCCAACG	TTTATACAGGGAGAGTTTAGAGAGTTC	275
crRNA4	19444199	19444177	CTTCCCGACGCTTCTTCTTCTTG	TTTG	CGACCCACACGCTGAAA	AGCTGCATGTGTAGTTGGTTAA	252
crRNA5	22438788	22438810	TGACACACAACCATGTGAAGGGC	TTTG	TGGCTCGGTAATGTGGTCT	CTGAACCCTAGCCTCATTACTC	296
crRNA6	19837043	19837065	TGCTCTCCCTTAGAACAACCTTG	TTTC	GATAATAGCTGCTTCTGCTGCT	TAGAGACAATACACAGTCACCCAA	243
crRNA7	22846142	22846164	ACTTGGTCGAAAGATATGAATGC	TTTG	GAATACGATATGCTCGGACATAC	TATGAGTGGGTATGTATATGGGTAG	290
crRNA8	20240356	20240378	GTCAAACTATAACAGCCTCACGG	TTTG	AATCTCATAGTCATAAAGTTAATTCGA	ATTTGAGTAGACGTTGCATGTTT	287
crRNA9	22919471	22919493	ATATTCAGCGGTTAACCTGAGCA	TTTC	TCATATTCACCAATTGGCTCA	ATAGCAGATGTAGAATCAACCACTT	298
crRNA10	20643957	20643935	GTCATAGTTATCTGCCAGGACAG	TTTC	CCTCTATCTCTAGTCAGTAGGCTTAC	CACAATCCCCTCAACCATC	222
crRNA11	23650018	23650040	AGCGATTGAGCAATGGAGGAATC	TTTC	GGAACTTACGTCGACAAACAA	ATGCTTAGATTTCAATTTATGAGC	291
crRNA12	21225289	21225311	GGTTGTCACACGGAGTCTCGATA	TTTG	CTCGACTTCAAATGAGCAAAGT	GCCAGATCCTATCTCACAAGG	241
crRNA13	24052150	24052128	GAGCAACTGGCGAATTTAGGTCT	TTTA	CCTTCTAATGGGTAAACACCAA	GTATGGGTTTGCACTTCGG	259
crRNA14	21627224	21627246	TAGCCATGTGATGCTTCGTTGAA	TTTG	GCCTAAATGGCGAGTCGT	AATGCCAGTCACACCGTATC	234
crRNA15	24252282	24252304	CTTGACTTAAATCCGTATGAGGC	TTTC	ATGTTGTATTCATGCTTGTTCG	GTCTGGTATTGGTACATTGATCC	240
crRNA16	24468261	24468239	GTGTTATCTGGTGTGACATGGTT	TTTA	CCAGAGACTAACCGATAAGGAGT	CGATTTGGGACACCAAGAG	174
